# Remote Vital Monitoring During Home Blood Transfusions in Japan Using Attendant‐Performed Vitals: A Pilot Feasibility and Safety Study

**DOI:** 10.1002/hsr2.72199

**Published:** 2026-03-24

**Authors:** Akinori Nishikawa, Sachiko Mitani, Akiko Miura, Hiroshi Akasaka

**Affiliations:** ^1^ Department of Hematology/Oncology Wakayama Medical University Hospital Wakayama Japan; ^2^ Division of Medical Informatics Wakayama Medical University Hospital Wakayama Japan; ^3^ Akasaka Clinic Kobe Japan

**Keywords:** alert, home blood transfusion, remote vital monitoring

## Abstract

**Background and Aims:**

Home blood transfusion is thought to be a promising solution for reducing the burden of hospital visits for elderly patients with hematologic diseases such as myelodysplastic syndrome and leukemia. However, safety concerns and reliance on nonprofessional attendants currently limit its widespread adoption in Japan. We evaluated feasibility (completion of scheduled measurement points per transfusion day) and operational safety (frequency of alerts and proportion leading to clinical actions) of intermittent vital sign monitoring with real‐time remote alerts during home blood transfusions in Japan.

**Methods:**

Five patients receiving home transfusions via Akasaka Clinic between December 2022 and February 2023 participated in the study. A digital tablet‐based system was preconfigured to connect with Bluetooth‐enabled vital sign devices (blood pressure monitor, thermometer, and pulse oximeter). Before transfusion, baseline vital signs were measured by the visiting physician; attendants then measured vitals at 15 and 60 min and at the end of transfusion. Data were automatically transmitted to physicians via the Heartline™ system. If preset thresholds were exceeded, alerts were triggered, which prompted professional medical intervention.

**Results:**

During the 30‐day trial period, red blood cell transfusions were performed 27 times (each consisting of 2 units), and platelet concentrate transfusions were performed 15 times (each consisting of 10 units). Measurement completion rates ranged from 38% to 94% (median 70% [IQR 53–88]). Alerts occurred on 12/30 days and totaled 12 alert events, corresponding to 28.6 alerts per 100 transfusions. Four of 12 alert events (33.3%) led to clinical actions (medication administration or initiation of home oxygen). Notably, intermittent measurement effectively identified clinically significant changes. Attendants, including elderly cohabitants, were able to operate the system with minimal burden.

**Conclusion:**

Intermittent, attendant‐performed vital sign monitoring with real‐time alerts enabled timely clinical responses and was feasible, even among elderly caregivers.

## 1 Introduction

As societies age, home healthcare is becoming increasingly important.　Regular blood transfusions are necessary for patients with hematologic diseases such as myelodysplastic syndrome and acute leukemia. However, the burden of frequent hospital visits for transfusions is growing for many patients and those who attend to them [1]. Elderly patients, for example, often require hospitalization due to difficulty in regularly traveling to the hospital. Home transfusions may offer a substantial benefit of reducing the burden of regular hospital visits for such patients. Nevertheless, home transfusions remain limited to a comparatively small number of facilities due to concerns about safety, drug management, and the handling of any complications or problems that might arise.

Ensuring safety is one of the most critical issues in relation to home transfusions. In Japan, a typical home transfusion procedure involves a medical professional visiting the patient's home to monitor for adverse reactions during the first 15 min of the transfusion and then leaving, and further monitoring is left to the attendant. Current guidelines require the presence of such an attendant to observe the patient's condition after medical personnel have left the home [2].

These attendants responsible for patient observation were reported in a nationwide survey in Japan to be typically clinic nurses, home nursing station staff, or family members, which suggests that adequately trained medical professionals are not always present. In this report, for example, over 80% of attendants were family members, which highlights the need to improve the quality of patient monitoring with the cooperation of nonprofessional caregivers [3].

In other countries, for example, Spain, home transfusions have reportedly been conducted by trained nurses, with assistance from trained family members or caregivers, and with a physician available on call [4, 5]. The patient's attendants nonetheless bear great responsibility, especially in recognizing complications and appropriately contacting medical personnel when necessary. We previously aimed to enhance patient safety and to reduce the burden on attendants by using remote vital sign monitoring systems that could alert medical staff to transfusion‐related complications [6]. Our findings indicated that simultaneous abnormalities in multiple vital signs could serve as effective indicators of such complications, such as increased pulse rate, decreased SpO₂, and increased respiratory rate. However, these systems required a certain level of proficiency in the setup and for the operation of the monitoring equipment, so we suggested that widespread adoption would be difficult. Additionally, the repeated need to install and retrieve equipment for each transfusion posed a significant burden [6]. Furthermore, continuous vital monitoring introduces challenges, such as interpreting fluctuations caused by patient movement or equipment adjustments. These limitations motivate consideration of an intermittent workflow based on scheduled vital measurements performed by attendants and supported by real‐time remote alerts, but evidence on its operational safety during home transfusions remains limited.

Therefore, we tested the feasibility (completion rates of scheduled measurement points per transfusion day) and operational safety (detection of changes, alert frequency, and resulting clinical responses) of an intermittent measurement workflow with real‐time remote alerts during home blood transfusions in Japan.

## 2 Materials and Methods

### 2.1 Study Design

Enrolled in this study were patients who received home blood transfusions through Akasaka Clinic (Kobe, Japan) between December 2022 and February 2023. This was a single‐center prospective pilot feasibility and safety study. Eligibility criteria were: patients aged ≥ 20 years and who provided informed consent; patients with a history of transfusions without severe adverse reactions; patients who understood and accepted the risks associated with home transfusions compared with those administered in medical institutions; and patients who had an attendant capable of measuring blood pressure, temperature, and SpO₂ using a pulse oximeter. Patients were excluded if the attendant was unable to reliably operate the devices or perform scheduled measurements, if adequate 4G connectivity was not available at the patient's home, or if the patient had any prior history of severe transfusion‐related complications requiring urgent medical intervention.

A visiting physician explained the study to each patient and attendant, and those who consented were included. The study was approved by the Wakayama Medical University Hospital Ethics Committee (Approval No. 3707, approved on November 21, 2022). Informed consent was obtained both orally and in writing from the patients and their families. Personal information was protected in accordance with relevant regulations, and all data were anonymized prior to analysis to ensure confidentiality.

## 3 Method

**Figure 1:**
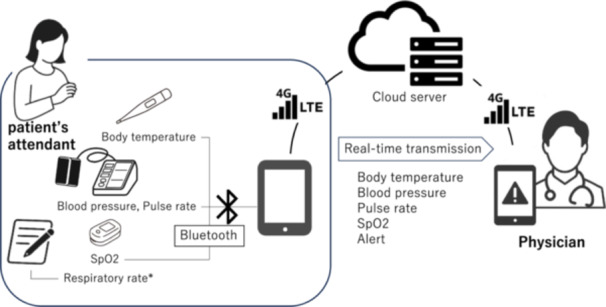
Heartline™ system. The system connects home‐use medical devices (including a blood pressure monitor, thermometer, and pulse oximeter) to a digital tablet device via Bluetooth. The iPad then transmits the measured vital signs to a cloud server using a 4G/LTE cellular connection. When the patient's attendant takes measurements using these devices, the data are automatically uploaded to the cloud and sent in real time to the smartphone of a physician at a remote location. * The respiratory rate measured by the SpO₂ monitor was not compatible with the current system, so it was manually recorded on a check sheet at the patient's home and was not used for real‐time remote monitoring in this study.

At each patient's home, where home blood transfusions were conducted, a digital tablet device (iPad, 9th generation, cellular model with 4G communication capability), a blood pressure monitor (NIPRO NBP‐1BLE), a thermometer (NIPRO NSM‐1BLE), and a pulse oximeter (Masimo MightySat R) capable of measuring respiratory rate (all Bluetooth‐enabled) were installed. These devices were each linked to the digital tablet device so that measured values were automatically recorded and transmitted via the internet to the attending physician's smartphone using the Heartline™ system version 3.0 (NIPRO Corporation, Osaka, Japan) (Figure 1). Bluetooth pairing was configured in advance and the devices were installed at the patient's home; therefore, re‐pairing was not required for each transfusion session. Under stable 4G connectivity, transmission latency from measurement to alert delivery was typically within ~1 s. The HeartLine system was not configured to retain system logs for pairing or transmission failures. During 4G connection loss, measurements were not buffered and thus were treated as missing for the corresponding time window.

The system allowed the setting of alert thresholds for each vital sign, and an alert was sent immediately to the physician's smartphone when any single measurement exceeded the preset limits. Before transfusion, a home care physician measured baseline vital signs (temperature, blood pressure, pulse rate, SpO_2_, and respiratory rate). After they left, the patient's attendant measured the same parameters at 15 min, 60 min, and upon completion of the transfusion. We defined measurement windows of ±15 min for the 15‐min and 60‐min time points (15 ± 15 min and 60 ± 15 min). If multiple measurements occurred within the same window, the first measurement was used for analysis (no duplicate measurements within a single window occurred in this study). If any scheduled measurement was missing, it was treated as incomplete (missing) for feasibility evaluation and was excluded from item‐level summaries; missing values were not imputed.

If complications were suspected, the patient or attendant was instructed to immediately measure vitals and contact the medical staff by telephone. Physicians could check vital data in real time via smartphone and respond appropriately, either by calling the home or making an emergency visit if necessary. In the absence of clinical symptoms suggestive of complications, attendants continued scheduled measurements.

Survey items included all vital signs recorded during transfusion, system‐generated alerts (e.g., abnormal pulse rate, SpO_2_, blood pressure), the patient's condition at the time of the alert, and actions taken in response.

Feasibility was assessed as the completion rate of four scheduled measurement points per transfusion day: before transfusion (baseline), 15 min after starting, 60 min after starting, and at the end of transfusion. For the 15‐min and 60‐min assessments, predefined tolerance windows of ±15 min were applied. Baseline and end‐of‐transfusion measurements were assessed as scheduled point measurements. A scheduled point was counted as complete only when all four parameters (respiratory rate, blood pressure, body temperature, and SpO₂) were recorded; otherwise, it was treated as incomplete (missing). In addition, we calculated the proportion of transfusion days in which at least 75% of the four scheduled measurement points were complete.

Operational safety was assessed as the number of alerts per 100 transfusion sessions, the number and proportion of alerts resulting in clinical actions (medication administration, initiation of home oxygen, physician home visit, or emergency request), and the occurrence of clinically significant changes. Clinically significant change was defined a priori as a vital sign abnormality that prompted physician assessment and resulted in a clinical action.

Data transmission complied with SSL/TLS 1.2; access required an ID and password, and access logs were available. Data were stored on the Heartline™ cloud server and could be viewed by authorized personnel (home transfusion physicians, home care nurses, researchers, and system administrators).

### 3.1 System Overview

As mentioned above, the installed digital tablet device was connected via Bluetooth to a thermometer, a blood pressure monitor, and a pulse oximeter. Attendants used these devices as they normally would, and then the measured data were automatically transmitted to the physician's device via a cloud server. The system required no special operations by the attendant, as data transmission occurred through routine measurement. Automatic transmission of respiratory rate data was still under development, so attendants were asked to record this value manually on a check sheet.

## 4 Results

Five patients receiving home blood transfusions through Akasaka Clinic provided informed consent and were eligible for inclusion in the study. An app for vital sign monitoring and alert notification was installed on the smartphones of three home transfusion physicians and home care nurses, enabling real‐time remote monitoring and prompt response to any complications.

Patient characteristics and transfusion information are summarized in Table 1. All met the inclusion criteria: there was no history of severe transfusion‐related complications in any of the included patients, and all were attended to by a capable attendant. Diagnoses included two cases of myelodysplastic syndrome, two of leukemia, and one of pure red cell aplasia. Patient ages ranged from 69 to 88 years, and all were unable to visit a hospital.

**Table 1:** Patient characteristics and transfusion information.

Pt. No	Age	Sex	Attendant	Disease	Days of transfusion	Number of red blood cells transfusions	Number of PC transfusions	Days with alerts
1	88	M	Son, daughter	Pure red cell aplasia, dementia	6	6	1	5
2	71	F	Husband	Acute leukemia	4	4	0	0
3	72	F	Husband	Myelodysplastic syndrome, diabetes	6	6	0	3
4	73	M	Wife	myelodysplastic syndrome, COPD	8	8	8	2
5	69	M	Wife	Acute leukemia, organizing pneumonia	6	3	6	2

*Note:* Days of transfusion: Number of days transfused with red blood cells, platelets, or both.

Abbreviation: PC, platelet concentrate.

Over the 30‐day observation period, abnormal vital alerts were generated on 12 days. A total of 27 red blood cell transfusions (all 2 units) and 15 platelet concentrate transfusions (all 10 units) were administered. Attendants included four cohabiting companions and one family member visiting an institutionalized patient from elsewhere.

At the start of the study, to avoid excessive false alerts, we applied provisional thresholds uniformly across all five patients. These were deliberately set with a wide range: SpO₂ between 90% and 100%, systolic blood pressure between 90 and 160 mmHg, heart rate between 50 and 100 bpm, and body temperature between 35.5°C and 37.5°C. Alerts were triggered when any measurement fell outside of these ranges.

Vital signs were assessed at four scheduled measurement points per transfusion day: before transfusion (baseline), 15 min after starting, 60 min after starting, and at the end of transfusion. Baseline measurements were obtained by the visiting physician, whereas the remaining three scheduled measurements were performed by the attendant. Respiratory rate was recorded manually, while the other vital signs (blood pressure, pulse rate, temperature, and SpO₂) were monitored remotely. Some scheduled measurements were missed because attendants forgot to measure temperature or because of equipment‐related problems. Using this four‐point definition, a total of 120 scheduled measurement points were expected over the 30‐day observation period (30 transfusion days × 4 points), of which 79 were complete, corresponding to an overall completion rate of 65.8%. Measurement completion rates by patient ranged from 38% to 94% (Table 2). Patient 5 showed the lowest completion rate, mainly due to the patient's declining condition and missed timing by the attendant.

**Table 2:** Completion of scheduled measurement points per transfusion day.

Pt. No	Attendant	Days of transfusion, *n*	Scheduled measurement points (4 per transfusion day), *n*	Completed measurement points, *n*	Completion rate, %	Days with ≥ 75% measurement completion, *n*	Days with ≥ 75% measurement completion rate, %
1	Son, daughter	6	24	17	70	4	67
2	Husband	4	16	15	94	4	100
3	Husband	6	24	21	88	5	83
4	Wife	8	32	17	53	4	50
5	Wife	6	24	9	38	1	17

After the initial phase of the study, we reviewed the frequency and content of alerts, along with the associated clinical symptoms. Based on this, we individually adjusted threshold values in cases where deviations were not associated with clinically significant events. For example, in Patient 1, who showed an increase in systolic blood pressure during transfusion without any serious adverse symptoms, we raised the upper limit to 170 mmHg. In Patient 3, whose baseline systolic blood pressure was naturally low, we reduced the lower threshold to 80 mmHg. In Patient 5, who had preexisting pneumonia and elevated temperature before transfusion, we adjusted the upper limit for body temperature to 38.0°C (Table 3). Alerts occurred on 12 of 30 days, including incidents of hypertension, low SpO_2_, and hypotension. For elevated blood pressure, antihypertensive medication was administered. For low SpO_2_　due to pneumonia exacerbation, oxygen therapy was provided. Other abnormalities, such as minor drops in temperature or systolic blood pressure, were within expected ranges and managed by observation. Alert thresholds were adjusted as needed according to each patient's baseline values. Patient 4, who had chronic obstructive pulmonary disease, had a transient SpO_2_ drop associated with movement and walking to the toilet. In total, 12 alert events occurred, corresponding to 28.6 alerts per 100 transfusions, and four events (33.3%) resulted in clinical actions (Table 4).

**Table 3:** Initial alert thresholds and patient‐specific adjustments.

Category	Vital sign	Initial threshold (standard baseline)	Adjusted threshold	Clinical justification for adjustment
All patients	SBP (systolic BP)	90–160 mmHg	—	Standard monitoring range
	PR (pulse rate)	50–100 bpm	—	Standard monitoring range
	SpO_2_	90%–100%	—	Standard monitoring range
	BT (body temp)	35.5°C–37.5°C	—	Standard monitoring range
Patient 1	SBP (upper)	160 mmHg	170 mmHg	Baseline hypertension
Patient 3	SBP (lower)	90 mmHg	80 mmHg	Baseline SBP consistently around 90 mmHg
Patient 5	BT (upper)	37.5°C	38.0°C	Persistent low‐grade fever due to an underlying disease

**Table 4:** Symptoms and treatment when a system alert is triggered.

Pt. No	Alert	Symptom	Treatment
1	Increased systolic blood pressure	No clinical symptoms	Oral drug administration
	Increased systolic blood pressure	No clinical symptoms	Oral drug administration
	Increased systolic blood pressure, increased pulse rate	No clinical symptoms	Improved with follow‐up
	Increased systolic blood pressure	Agitation due to dementia	Normal value on retest
	Increased systolic blood pressure	No clinical symptoms	Oral drug administration
3	Decreased systolic blood pressure, hypothermia	No clinical symptoms	Normal value on retest
	Decreased systolic blood pressure, hypothermia	No clinical symptoms	Follow‐up
	Hypothermia	No clinical symptoms	Follow‐up
4	Decreased systolic blood pressure, decreased SpO_2_	Pneumonia, after walking to the toilet	Improved with follow‐up
	Decreased SpO_2_	After walking to the toilet	Improved with follow‐up
5	Decreased pulse rate	No clinical symptoms	Follow‐up
	Elevated body temperature, decreased SpO_2_	Pneumonia, shortness of breath	Start home oxygen

Simultaneous increases in respiratory rate supported that this was not a measurement error. Episodes with simultaneous vital sign abnormalities were considered significant changes in condition, and we suggest this highlights the importance of including respiratory rate in assessments.

## 5 Discussion

In this study, we aimed to evaluate the feasibility and safety of home blood transfusion monitoring with the cooperation of patient attendants using a simple, low‐burden remote vital monitoring system.

Most of the attendants were cohabiting companions or spouses, likely to be of similar age to the patients. By preconfiguring the digital tablet system, these attendants needed only to operate familiar devices, which enabled even elderly caregivers to perform measurements without added burden. Meanwhile, the check sheet used to record respiratory rate helped prevent missed measurements, even in home environments with limited medical supervision. The alert function allowed medical staff to be promptly notified of abnormal vital signs, enabling timely and appropriate interventions. However, determining appropriate alert thresholds for each patient posed a challenge. Initial settings were uniform, with adjustments made as necessary based on individual baselines. All adjustments were based on clinical judgment and observed physiological responses, so no algorithmic or automated assistance was used in this process. A potentially useful system improvement would be to develop algorithms or automation to support patient‐specific threshold adjustment. However, substantial physiological variability in elderly individuals and in patients with complex conditions may limit the straightforward application of such automation.

Continuous remote vital monitoring system in home care has been examined in previous studies. Although featuring real‐time data acquisition, there are often motion artifacts and false alarms, and there is the burden of constant device management by caregivers [6, 7]. In contrast, our approach, which is based on supervised, intermittent vital sign measurements, may have reduced the likelihood of data misinterpretation and allowed re‐evaluation when abnormal values were detected. Furthermore, the simple operation may have reduced user fatigue and may have improved acceptability among elderly caregivers. Similar user‐centered, low‐complexity designs have been shown to promote successful adoption of health technologies in aging populations [8].

Our system also demonstrated potential for posttransfusion monitoring or observing nontransfusion‐related complications, such as pneumonia. This aligns with current Japanese home transfusion guidelines, which recommend continued observation after transfusion to detect delayed severe complications such as transfusion‐related acute lung injury or transfusion‐associated circulatory overload [2]. Notably, in one of our patients (Patient 2, Table 1), no alerts were triggered during the transfusion itself, but there was elevated temperature and pulse rate recorded the following day. This enabled early detection of cerebral hemorrhage, and it illustrates the broader applicability of the system in tracking health deterioration beyond transfusion‐specific events.

Structured home transfusion programs involving trained nurses and family caregivers under physician supervision have been implemented in some countries, for example, in Spain [4, 5]. However, the operational models in these programs often rely on specialized staff or sophisticated logistics. Our findings provide a practical perspective by showing that even elderly, nonprofessional caregivers (most of our attendants were the patients' spouses) were able to participate in remote safety monitoring when supported by preconfigured, automated systems. Among our participants, the main reasons for imperfect measurement completion rates were the patient's deteriorating condition, which made scheduled measurements difficult, as well as device malfunctions and omissions caused by attendant inattention. To address this, features such as voice‐based measurement reminders could be considered in future system improvements to help reduce missed measurements.

Compared with continuous remote monitoring systems, which often involve wearable sensors or constant data streaming, the intermittent model used in this study showed several practical advantages. For instance, it is comparatively less susceptible to motion artifacts, and it does not require continuous caregiver oversight or technical maintenance. In terms of cost, the use of commercially available devices (digital tablet device, thermometer, blood pressure monitor, pulse oximeter) and cloud‐based transmission allows for low initial investment and easier scalability. Usability was likely supported by limiting required operations to basic measurements with familiar devices, an approach that may be particularly important for elderly attendants.　 Continuous systems may offer finer temporal resolution, but our findings indicate that supervised, intermittent monitoring can serve as a practical and cost‐effective alternative in the home setting. A qualitative comparison of intermittent and continuous monitoring modalities is summarized in Table 5.

**Table 5:** Qualitative comparison of monitoring modalities.

Feature	Intermittent monitoring (current system)	Continuous monitoring (wearable/bedside)
Hardware and implementation cost	Low: Utilizes consumer‐grade bluetooth devices.	High: Requires specialized medical‐grade sensors and infrastructure.
Caregiver involvement	Manual measurements required at scheduled intervals.	Hands‐off data collection, but requires sensor management and skin care.
Data accuracy & artifacts	High: Measured while the patient is at rest; minimal motion artifacts.	Variable: High susceptibility to motion artifacts and signal noise.
Alarm reliability	Alerts are usually clinically significant and actionable.	High rate of false alarms, often leading to “alarm fatigue.”
Scalability	High: Easily deployed using existing 4G/LTE networks and simple devices.	Moderate: Requires complex setup and dedicated technical support.
Patient acceptability	High; nonintrusive and allows for normal daily activities.	Moderate to Low; constant attachment can cause discomfort or anxiety.

All attendants in this study received verbal instructions and demonstration from the visiting physician during the initial home visit that covered device use and the timing of measurements. No formal usability survey was conducted this time, but informal feedback was collected during follow‐up visits. Future studies should incorporate structured usability assessments using validated instruments (e.g., the System Usability Scale) and objective measures such as time to complete the scheduled measurements and the frequency of usage errors or missed measurements. Additionally, expanding such monitoring to cover posttransfusion observation periods, such as the 24 h following transfusion, could improve detection of delayed reactions and align with best hemovigilance practices.

This study has certain limitations that require careful consideration, most notably the small sample size (*n* = 5), which restricts the statistical power and limits the generalizability of the findings. As a pilot investigation, our primary aim was to assess the feasibility and safety of the system rather than to derive statistically robust conclusions. Accordingly, these results should be interpreted as hypothesis‐generating.

The findings are most applicable to home transfusion settings in which attendants are available, device use is preconfigured, stable 4G/LTE connectivity is available, and care is delivered within an organized home‐care framework with physician oversight. Accordingly, generalization to other settings should be made with caution. Transferability to other systems is likely to depend less on the specific Heartline™ platform itself and more on whether comparable core functions—automatic data transmission, real‐time alerting, remote review, and timely professional response—can be implemented. In lower‐acuity settings, this workflow could potentially be adapted to simpler alert‐driven models, including those based on smartwatch‐derived signals, although the range of measurable vital parameters would differ from that in the present study. However, such broader applications were not evaluated here and would require separate validation before clinical adoption.

To validate the broader applicability and to refine the monitoring framework, future studies involving larger and more diverse patient populations across multiple institutions are warranted. Such studies would allow for more rigorous evaluation of clinical outcomes, alert threshold optimization, and system usability across varied home care settings.

In conclusion, this study introduced an apparently safe and practical remote monitoring system for home blood transfusions, facilitated by patient attendants. When attendants can use standard devices, this system enabled effective data transmission, including in elderly caregivers. The supervised, intermittent monitoring model offers a pragmatic alternative to continuous systems and could help address barriers to the wider implementation of home transfusions in aging societies. Future work should focus on scaling this approach, integrating predictive analytics, and establishing structured caregiver education programs to ensure sustainable operation and patient safety.

## Author Contributions

**Akinori Nishikawa:** conceptualization, methodology, data curation, investigation, formal analysis, writing – original draft. **Sachiko Mitani:** investigation. **Akiko Miura:** investigation. **Hiroshi Akasaka:** resources. All authors have read and approved the final version of the manuscript. The corresponding author Akinori Nishikawa had full access to all of the data in this study and takes complete responsibility for the integrity of the data and the accuracy of the data analysis.

## Conflicts of Interest

The authors declare that they have no competing interests.

## Transparency Statement

The lead author Akinori Nishikawa affirms that this manuscript is an honest, accurate, and transparent account of the study being reported; that no important aspects of the study have been omitted; and that any discrepancies from the study as planned (and, if relevant, registered) have been explained.

## Data Availability

The datasets used and analyzed during the current study are available from the corresponding author on reasonable request.
